# Feasibility and Preliminary Efficacy of a Telerehabilitation Intervention for Diastasis Recti Abdominis—A Pilot Study

**DOI:** 10.3390/healthcare13172224

**Published:** 2025-09-05

**Authors:** Anastasia Skoura, Maria Antoniou, Nikolaos Thanatsis, Dimitra Tania Papanikolaou, George Tsirogiannis, Elena Drakonaki, Evdokia Billis

**Affiliations:** 1 Laboratory of Clinical Physiotherapy and Research, Department of Physiotherapy, School of Health Rehabilitation Sciences, University of Patras, 26504 Patras, Greece; mariaantoniou1997@yahoo.gr (M.A.); dpapanikolaou@upatras.gr (D.T.P.); billis@upatras.gr (E.B.); 2 School of Medicine, University of Patras, 26504 Patras, Greece; nikthanatsis@upatras.gr; 3 Department of Food Science and Technology, University of Patras, 26504 Patras, Greece; gtsirogianni@upatras.gr; 4 Anatomy Department, Medical School, University of Crete, Crete, 70013 Heraklion, Greece; drakonaki@yahoo.gr

**Keywords:** telerehabilitation, diastasis recti, postpartum rehabilitation, usability, satisfaction

## Abstract

**Background**: Diastasis recti abdominis (DRA) is a common postpartum condition typically managed with rehabilitation exercises. Given its high prevalence and postpartum barriers in attending in-person sessions, telerehabilitation may offer a feasible alternative. This small pilot study evaluates the preliminary effectiveness and user satisfaction of a 12-week telerehabilitation exercise program for women with postpartum DRA. **Methods**: Parous women with DRA participated in a 12-week trunk stabilization program, including synchronous and asynchronous sessions, from April 2024 to May 2025. The primary outcome was satisfaction (Telehealth Usability Questionnaire—TUQ_Greek and two additional custom-made questions). Secondary outcomes included inter-recti distance (IRD), trunk muscle endurance tests, body image (BISS_Greek), and adherence to exercise. **Results**: Thirteen participants aged 37.54 ± 5.49 completed the pilot intervention. Satisfaction was high (TUQ_Greek = 6.28 ± 0.60), with 84.62% (11/13 subjects) rating telerehabilitation as very satisfactory. Statistically significant reductions in IRD were observed at 2 cm (large effect, d = 1.00; 95% CI [0.12 to 0.47]) and 5 cm (large effect, d = 0.81; 95% CI [0.08 to 0.58]) above the umbilicus (*p* < 0.05). Post-intervention, most trunk muscle endurance tests improved significantly (*p* < 0.05) at 4 and 12 weeks (large effect, η^2^ = 0.44 to–0.56). Body image (BISS_Greek) also improved post-intervention (*p* < 0.05, medium to large effect, d = −0.73; 95% CI [(–1.75 to –0.16]). Mean adherence reached 71.37%. **Conclusions**: This small pilot supports the feasibility and acceptability of a telerehabilitation program as well as its effectiveness in improving key clinical outcomes. However, since this was a small pilot, generalizability might be limited by the small sample size and should be confirmed in larger studies.

## 1. Introduction

Diastasis recti abdominis (DRA) is a trunk dysfunction caused by the separation of the two rectus abdominis muscles along the linea alba due to its stretching and thinning. It typically develops during pregnancy, with a prevalence ranging from 66 to 100% in the third trimester, and may persist postpartum, affecting 39–45.4% of women in the first six months [1,2], and up to 32.6% by twelve months postpartum [2]. It is driven by hormonal changes, increased intra-abdominal pressure from fetal growth, and organ displacement [3,4]. Symptoms may include abdominal muscle weakness, core instability, lower back and pelvic pain, pelvic floor dysfunction, and a negative body image, all of which can impact maternal well-being and daily function [5,6,7].

Physiotherapy, and specifically structured therapeutic exercise programs, are recommended as the first-line treatment for DRA [8,9]. However, despite the reported positive effects of exercise on physical and emotional health, new mothers often face challenges with participating in regular exercise, especially in in-person exercise sessions, due to difficulty balancing childcare, household responsibilities, and work [10]. A few studies on asynchronous telerehabilitation methods for DRA (i.e., mobile application- or video-based programs) [11,12] have provided encouraging results for inter-recti distance (IRD) reduction and related outcomes; there is only one though examining adherence and participant satisfaction [12].

Meanwhile, synchronous telerehabilitation, that is, rehabilitation in real time, with concurrent observation, interaction, and supervision from the healthcare professional, has been explored to date by only one RCT [13], which utilized real-time teleconferencing, reporting significant improvements in IRD and in some functional and quality of life areas. Yet, the study’s insufficient duration (only six weeks), absence of adherence and participant satisfaction reporting, and inclusion of relatively “mild” cases of DRA limit the applicability of its findings. Furthermore, existing studies lack blended synchronous and asynchronous delivery, as well as validated usability measures to evaluate acceptance or satisfaction. These gaps highlight the need for further research to establish the optimal design of telerehabilitation programs, their comparative effectiveness against in-person interventions, long-term outcomes, applicability in women with more persistent or at least “moderate” DRA, patient satisfaction, and adherence.

This small pilot study is, to our knowledge, the first to evaluate a 12-week, blended synchronous–asynchronous telerehabilitation program for women more than 6 months postpartum with moderate DRA, using validated usability measures alongside clinical and psychosocial outcomes. This study aimed to evaluate feasibility, patient satisfaction, and preliminary effectiveness and hypothesized that participants would report high satisfaction and usability scores, and that the program would lead to improvements in IRD, trunk muscle endurance, and body image.

## 2. Materials and Methods

### 2.1. Study Design

This was a prospective, single-arm intervention pilot, intended to inform the design of larger randomized controlled trials. Ethical approval was given by the Ethics Committee of the University of Patras (Number 16331/29-01-2024), and the entire work was carried out in accordance with the Code of Ethics of the World Medical Association (Declaration of Helsinki) for experiments involving humans. This study has been registered on ClinicalTrials.gov (NCT07017309).

### 2.2. Participants

This study was conducted between March 2024 and May 2025. Participants from the broader region of Achaia (mainland Greece) were invited to participate in this study through the distribution of informative leaflets and posters across the university campus and university hospital areas. Each participant gave their informed consent before their enrollment.

Participant inclusion criteria were (a) female volunteers aged between 18 and 50 (b) with diagnosed DRA (inter-recti distance ≥ 2.8 cm) [14], (c) at least 6 months (to avoid the confounding natural resolution of DRA during early postpartum) and up to 5 years post-birth, (d) able to voluntarily contract the transversus abdominis (TrA) and pelvic floor muscles (PFMs), the key muscles contracted to tense the linea alba, as confirmed with ultrasonographic biofeedback training [9,15,16], and (e) experienced with digital technology and having access to a smartphone, tablet, or computer with a camera and internet connection. Participants were excluded from this study if they had (a) undergone recent abdominal surgery (excluding cesarean section), (b) serious musculoskeletal, neurological or respiratory conditions limiting exercise participation, (c) connective tissue disorders affecting the linea alba, or (d) a body mass index (BMI) ≥ 30 kg/m^2^, as higher adiposity could hinder accurate measurement and imaging of the abdominal wall [17].

### 2.3. Procedures

Participants who met the eligibility criteria and provided informed consent received a baseline assessment and in-person training during the same appointment to ensure they could correctly activate key muscle groups through palpation and ultrasound biofeedback [15,16]. All ultrasound measurements and ultrasonographic biofeedback muscle training were conducted by an experienced physiotherapist who acted as an independent examiner, blinded to the intervention. The reliability of this physiotherapist’s measurements was rated as very good in a previous study [18]. The same independent examiner, assisted by another physiotherapist experienced and familiar with current procedures, administered the questionnaires and assessed muscle endurance using functional motor control tests.

### 2.4. Intervention

All participants completed a 12-week telerehabilitation program targeting DRA, led by two physiotherapists specializing in pelvic health. The intervention was delivered in small groups of 3–4 women, with three scheduled sessions per week, two synchronous supervised sessions via teleconference using Zoom Video Communications platform (Zoom Video Communications, CA, US), and one asynchronous (unsupervised) session using pre-recorded videos. Our rationale was to facilitate women’s attendance, by offering one session to be undertaken in their own time, while maintaining a good level of synchronous supervision and monitoring. Participants were not instructed to perform any additional independent exercise outside the scheduled sessions but were encouraged to adopt abdominal wall protection strategies during daily activities (lifting, pushing, pulling).

The 12-week exercise protocol was standardized for all participants and delivered according to a specific schedule (see Supplementary Material Table S1). The physiotherapist providing the exercise program received full training on the protocol from the lead physiotherapist before the intervention, to ensure consistency. Both were present during all synchronous sessions, one delivering the exercises, while the other monitored technique, recorded adherence, resolved technical issues, and ensured protocol fidelity. Minimal adaptations to exercises (modifications in leverage arm, or plank on knees if unable to maintain on feet) were permitted only when participants could not safely perform the standard version, following predefined modification guidelines.

Synchronous sessions. Supervised sessions were scheduled based on participant availability; however, if two consecutive supervised sessions were missed, participants were contacted to reschedule at a convenient time. If rescheduling was not possible, they were encouraged to attend the corresponding asynchronous session available on the program platform to maintain the target exercise frequency. Ongoing online supervision, education, and feedback ensured correct technique and exercise progression.

Asynchronous sessions. All pre-recorded exercise videos and supplementary educational materials were delivered through a blended learning platform (Google Classroom platform), customized specifically for the program.

Exercise composition. The exercise program combined PFM training, TrA activation, diaphragmatic breathing, stabilization, strength training, and functional exercises, following the latest recommendations in the field [9,19]. Exercise duration was 20–45 min, with gradual progression in difficulty, complexity, repetitions, and load. Intensity was monitored using the 10-point Rate of Perceived Exertion (RPE) scale, targeting a moderate level (5–6), and adjusted as needed.

### 2.5. Primary Outcome

Primary outcomes included usability and participant satisfaction, assessed using the Telehealth Usability Questionnaire (TUQ), along a single-item rating and open-ended question. TUQ is a validated tool comprising 21 items assessing six domains: (i) usefulness, (ii) ease of use and learnability, (iii) interface quality, (iv) interaction quality, (v) reliability, and (vi) satisfaction and future use. It is primarily developed for synchronous telehealth sessions involving real-time video conferencing between patients and healthcare providers [20], and it was therefore, deemed appropriate for this study design, which was predominantly based on synchronous telerehabilitation sessions. TUQ items are rated on a 7-point Likert scale, from “1: strongly disagree” to “7: strongly agree”, and higher scores indicate greater usability [20]. The overall TUQ score is computed by adding and averaging the scores of all individual items, with the lowest possible mean being 1 and the highest being 7. TUQ has been translated and culturally adapted into Greek (TUQ_Greek) following established forward–backward translation procedures [21]. User satisfaction was further complemented with a closed-ended question, derived from the existing literature [22,23], asking participants to rate their satisfaction with the telerehabilitation program on a 5-point Likert scale, from “not at all satisfied” to “very satisfied.” Additionally, an open-ended question invited participants to compare the level of support and guidance received during telerehabilitation sessions with that of in-person sessions, providing some qualitative insights.

### 2.6. Secondary Outcomes

Our secondary, exploratory investigations included DRA-related outcome measures such as IRD, trunk muscle endurance, body image, and exercise adherence. All assessment sessions took place at the same time of day for each participant.

Inter-recti distance (IRD). IRD was assessed using a real-time 2D diagnostic ultrasound imaging unit (Versana Active™, GE Healthcare, Illinois, US) at three locations: at 2 cm and 5 cm above the umbilicus and at 2 cm below the umbilicus, in a supine, relaxed position with knees flexed [14]. The ultrasound probe was held perpendicular to the abdominal wall and with minimal pressure, following our previously published methodology [18], where measurement reliability was rated as very good. A linear transducer set at 10 MHz in B-mode was used to capture images at the end of exhalation. IRD was measured using digital calipers provided by the ultrasound device’s built-in measurement system (See Supplementary Material-Figure S1).

Trunk muscle endurance tests. Four established tests [24] were performed, timed for maximum duration: the abdominal curl-up test, front plank, left and right side planks, and McGill’s Trunk Flexor Endurance Test [13,25,26]. Participants held each position for as long as possible while maintaining proper form. Tests ended when trunk alignment was lost and shoulders/hips dropped to the ground, to ensure a consistently correct execution. A 2 min resting period was included between tests.

Body image. Body image perception was assessed using the Body Image State Scale (BISS), a self-reported questionnaire created by Cash et al. (2002) [27] and cross-culturally adapted in Greek [28]. BISS_Greek consists of six statements related to how individuals perceive their physical appearance, body size and shape, weight, feelings of physical attractiveness, as well as current feelings about appearance compared to usual perceptions. Responses are rated on a 9-point bipolar Likert scale, with total scores ranging from 1 to 9 (higher scores indicating a more positive body image perception) [27].

Adherence. Participant adherence was tracked using custom-made exercise diaries developed by the research team, recording the number and type (remote supervised, or video-based) of weekly exercise sessions. Participants were encouraged to complete two supervised and one video-recorded exercise sessions per week. Attendance for supervised online sessions was documented by the physiotherapists, while completion of video-recorded sessions was self-reported by participants weekly. By study design, at least 67% adherence (at least 24 sessions) was required for inclusion in the final analysis, regardless of whether these were synchronous or asynchronous. To support adherence, participants who missed two consecutive supervised sessions were contacted to reschedule the missed session at a convenient time. If a mutually suitable time could not be arranged, participants were encouraged to complete the corresponding pre-recorded exercise session available on the program platform to maintain at least two sessions per week. Exercise adherence was assessed in two ways: as the proportion of completed sessions out of the total prescribed 36 sessions (total program adherence), and as the percentage of completed supervised (target: 24 sessions) and video-recorded (target: 12 sessions) sessions, respectively.

### 2.7. Statistical Analysis

Data were analyzed using IBM SPSS Statistics version 28.0.1.0. Descriptive statistics were applied to demographic and outcome data. The TUQ_Greek responses were analyzed using mean, standard deviation, minimum–maximum, median, and interquartile range for each domain. Normality was assessed with the Shapiro–Wilk test. Paired *t*-tests were used to compare pre- and post-intervention IRD and BISS_Greek scores. To examine changes in trunk muscle endurance over time, a repeated measures analysis of variance (ANOVA) was performed. Assumptions of sphericity were assessed using Mauchly’s Test of Sphericity. As sphericity was not violated (*p* > 0.05), standard repeated measures ANOVA results were reported. Statistical significance was set at *p* ≤ 0.05. Adherence data were descriptively analyzed. As this was an exploratory, single-arm pilot study, no formal multiplicity correction was applied. *p*-values for secondary and exploratory outcomes are presented unadjusted and should be interpreted with caution. Effect sizes for clinical measurements and body image outcomes were calculated using Cohen’s d for paired samples, along with 95% confidence intervals (CIs). Cohen’s d values were interpreted according to established thresholds, with 0.2 indicating a small effect, 0.5 a medium effect, and 0.8 or higher a large effect. For repeated measures ANOVA analyses, partial eta squared (η^2^) was used to estimate effect size according to established thresholds, ~0.01 indicating a small effect size, ~0.06 a medium effect size, and ~0.14 a large effect size.

## 3. Results

### 3.1. Participant Characteristics

**Figure 1:**
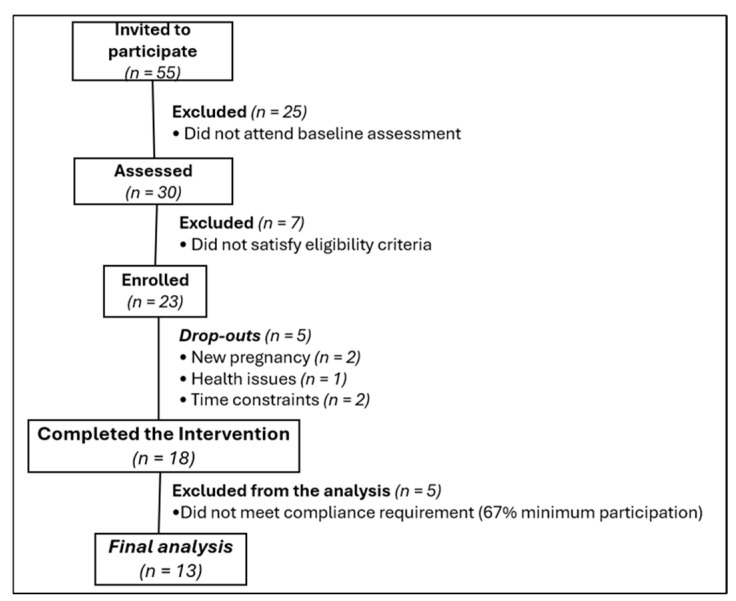
Flow chart of study participants from invitation to final analysis.

A total of 55 individuals initially responded to the study invitation. Figure 1 depicts the flow of participants through this study. Ultimately, data from 13 participants were included in the analysis. Participant demographic characteristics are presented in Table 1.

### 3.2. Primary Outcome—Usability and Participant Satisfaction

The overall mean score on the study primary outcome measure, TUQ_Greek, was 6.28 ± 0.60. Mean scores, minimum–maximum, median scores, and interquartile ranges (IQRs) in each subdomain are presented in Table 2. All participants responded to the closed-ended satisfaction question regarding their experience with telerehabilitation, with 84.62% (n = 11) reporting that they were “very satisfied” and 15.38% (n = 2) reporting that they were “satisfied” with the telerehabilitation program. Furthermore, all participants provided feedback to the open-ended question, which was clustered into four main categories: Zoom as an effective alternative when in-person sessions were not feasible (5/13; 38.5%), comparable support between telerehabilitation and in-person sessions (6/13; 46.2%), perceived advantages of in-person sessions (6/13; 46.2%), and preference for a combined approach (2/13; 15.4%). Representative responses are presented in the Supplementary Material.

### 3.3. Secondary Outcomes

Results for secondary outcomes are presented as exploratory analyses.

#### 3.3.1. Inter-Recti Distance (IRD) and Body Image

At 2 cm and 5 cm above the umbilicus, a statistically significant reduction was observed post-intervention (*p* < 0.05, 95% CI [0.12 to 0.47] and [0.08–0.58]), demonstrating large effect sizes (Table 3). At 2 cm below the umbilicus, the mean IRD decreased post-intervention, without reaching statistical significance (*p* > 0.05, 95% CI [−0.24 to 0.66]), and with a small effect size. The mean body image score on BISS_Greek significantly increased from baseline to the end of the intervention (*p* < 0.05, 95% CI [–1.75 to –0.16]), with a medium to large effect size.

#### 3.3.2. Trunk Muscle Endurance

Overall, mean score improvements were detected in all trunk endurance tests as the intervention progressed. Both left- and right-side planks and McGill’s Trunk Flexor Endurance test yielded statistically significant differences between baseline and end of intervention (at Week 12), with large effect sizes (Table 4). In addition, McGill’s Trunk Flexor Endurance test scores improved significantly at Week 4.

#### 3.3.3. Exercise Adherence

Overall mean adherence scored 71.37 ± 3.65%. Supervised (synchronous) telerehabilitation yielded 95.83 ± 6.80% adherence, while unsupervised (asynchronous) telerehabilitation adherence was 21.79 ± 13.41%.

## 4. Discussion

This study demonstrates high levels of usability and satisfaction with this DRA telerehabilitation program, alongside high adherence and preliminary improvements in clinical outcomes such as IRD, trunk function, and body image. Indeed, telerehabilitation has become an innovative and effective option in recent years, particularly following the COVID-19 pandemic. For DRA management, where exercise is the first-line treatment, telerehabilitation offers new mothers access to structured therapeutic exercise programs while minimizing barriers to in-person attendance.

While previous DRA telerehabilitation studies [11,12,13] have focused on clinical outcomes, patient experience, service feasibility, and satisfaction are equally important indicators of healthcare quality, influencing long-term acceptance. To our knowledge, this is the only study in the field to prioritize system usability and participant satisfaction as the primary outcomes and to use a validated questionnaire (TUQ_Greek) in this population. Both the TUQ_Greek and the closed-ended satisfaction item, which was adapted based on the literature [23], documented high acceptance and excellent system usability. Our findings are in line with previous studies employing TUQ for the assessment of online physiotherapy interventions across other patient populations [29,30,31].

Our primary outcomes showed that participants appreciated the convenience, accessibility, and time-saving benefits of remote physiotherapy sessions. The TUQ subscales receiving the highest score were the “ease of use”, indicating that the system was user-friendly, as well as the “satisfaction and future use” subscale, showing strong user engagement and willingness to continue using the online system. Qualitative feedback from the open-ended question reinforced these results, with nearly half of participants reporting that online support was equivalent to in-person care, while others highlighted the importance of blended approaches (see Supplementary Material Table S2). This preference for in-person care was further documented in the TUQ lowest-rated question concerning the acceptability of telehealth as a healthcare delivery method (question 19). “Interface quality” and “interaction quality” also scored highly, with users reporting pleasant, understandable interactions and effective communication with therapists. Real-time guidance was particularly valued, with nearly half of the participants reporting that the quality of support during remote sessions was equivalent to in-person care (question 15). The “usefulness” subscale score reflected agreement that telerehabilitation saved time and improved access; however, the lowest-rated item in this subscale (question 3) concerned whether all healthcare needs were met, highlighting some unmet expectations. “System reliability” had the lowest score, likely due to occasional technical issues or insufficient error messaging. High overall satisfaction was also reported in the closed-ended question, with 84.62% of participants reporting being “very satisfied” and 15.38% “satisfied”.

Secondary, exploratory outcomes indicated that the DRA telerehabilitation program also produced preliminary improvements in clinical parameters. In agreement with previous studies on DRA telerehabilitation [11,12,13], the results showed a statistically significant reduction in IRD post-intervention, at two out of the three measurement locations: 2 cm and 5 cm points above the umbilicus (*p* < 0.05, 95% CI [0.12 to 0.47 and 0.08 to 0.58]), although our observed IRD reduction was generally smaller. However, Leopold et al. [12] in a comparable 12-week protocol provided in asynchronous mode reported a similar reduction below the umbilicus (0.26 cm), consistent with our findings. Likewise, traditional in-person stabilization programs have reported reductions of similar magnitude, such as 0.24 cm at 4.5 cm above the umbilicus [32] and 0.20 cm at 2 cm below the umbilicus [33]. While a universally accepted minimal clinically important difference (MCID) for ultrasonographic IRD measurement has not been established, the smallest detectable difference (SDD), which quantifies a “real” change beyond measurement error, serves as a valuable indicator of clinical significance. Based on previous investigations of our research team [18], the SDD for IRD measurements at 3 cm above the umbilicus was 0.50 cm and for IRD measurements at halfway the distance between the xiphoid process and umbilicus was 0.55 cm. While our observed mean reductions of 0.29 cm and 0.33 cm, respectively, were statistically significant, they did not exceed the SDD threshold. This suggests that the observed changes, while statistically significant, may be subtle and warrant further investigation in a larger study to determine their clinical relevance. The reduction in IRD observed at 2 cm below the umbilicus was not statistically significant (*p* > 0.05, 95% CI [–0.24 to 0.66]), a finding aligned with previous RCTs [25,34], possibly attributed to the different anatomical and mechanical properties of the linea alba below the umbilicus; in this area, the linea alba displays greater elasticity, narrower width, lower mechanical tension, and limited support from surrounding tissues [4], potentially explaining the reduced responsiveness to exercise interventions. Nevertheless, a small effect was observed (Cohen’s d = 0.30, 95% CI [–0.29, 0.87]), indicating a trend towards a meaningful reduction in IRD at this location.

Unlike most DRA rehabilitation studies [9], this study included women at least 6 months postpartum. This was undertaken to reduce any potential bias due to the natural resolution of DRA, which according to the literature, usually occurs within the first 8 weeks after childbirth [3,35,36], but continues with possible changes up to 6 months [1]. Thus, this was considered an additional strength of the study.

Additionally, improvements (*p* < 0.05) were observed in the side planks and McGill’s trunk flexor endurance test from baseline to post-intervention, as well as between baseline and week 4 in McGill’s test, with large effect sizes, indicating large and clinically significant improvements. While the abdominal curl-up and front plank tests did not reach statistical significance, both showed large effect sizes, demonstrating clinically meaningful improvements over time. These findings are consistent with previous research on DRA rehabilitation, whether online or face-to-face [13,25], though earlier studies entailed shorter intervention periods, provided only pre- and post-intervention measurements, and included no additional interim assessments, as in this study. The statistically significant improvement reported in body image score (*p* < 0.05, 95% CI [–1.75 to –0.16]) was also an important finding. The effect size of the change was medium to large, meaning the difference was also clinically meaningful. That outcome was intentionally included in this study due to the strong association between DRA and negative body image, low self-esteem, and mental health issues [37,38].

Furthermore, this study reported a high overall participant adherence to the telerehabilitation program, reaching a total rate of 71.37%. Leopold et al. [12] reported a similar adherence rate (72%) during a 12-week DRA telerehabilitation program, though this program involved non-supervised, video-recorded sessions. A key novel contribution of our study is the blended design of synchronous and asynchronous components within the same telerehabilitation program. Interestingly, in our study, synchronous telerehabilitation yielded the highest adherence (95.83 ± 6.80%), corresponding to at least 21 out of 24 completed sessions, as opposed to our asynchronous sessions, which showed a much lower engagement (21.79 ± 13.41%). This indicated that participants only completed 1–4 video-recorded sessions throughout the program, which implies better compliance with supervised online sessions that encourage motivation, accountability, active supervision, and engagement. Our program’s philosophy in combining two synchronous and one asynchronous weekly sessions was to facilitate women’s attendance by offering one session to be undertaken in their own time, while maintaining a good level of synchronous supervision and monitoring. Indeed, participant feedback throughout this study indicated that real-time supervision by physiotherapists allowed immediate feedback, personalized guidance, continuous monitoring, and enhanced safety during exercise. Moreover, group-based teleconference sessions fostered stronger patient–provider relationships and a sense of community. The program’s group structure was intentionally designed to promote feelings of belonging, peer motivation, social interaction, and shared goals, factors that often contribute to higher adherence and engagement with telerehabilitation [39]. However, this discrepancy between synchronous and asynchronous adherence could suggest that unsupervised asynchronous sessions may be more difficult to complete consistently, particularly given the time constraints and competing demands typical in the postpartum period. The findings indicate that future telerehabilitation programs should account for postpartum women’s unique needs in their design. Asynchronous components certainly merit further discussion and may need to be simplified and more time-efficient, supplemented with motivational strategies, or integrated into a hybrid approach to promote and sustain engagement.

Verbal queuing and detailed instructions provided during synchronous sessions were also valued by participants, as recorded by their responses on the TUQ, further indicating that real-time teleconferencing was perceived as equivalent to in-person care and was probably favored over asynchronous sessions. Despite having received extensive in-person training during their baseline evaluation, participants often remained online after sessions to ask questions or clarify complex exercises. This is particularly common with deep trunk and pelvic floor rehabilitation exercises, as women tend to be unsure of their muscle contractions or body alignment as they acquire more awareness and control over the muscles. Interestingly, despite growing interest in telerehabilitation, few research reports consider synchronous modes of telerehabilitation [13], even though real-time feedback from healthcare professionals seems to be necessary with postpartum rehabilitation exercises. Thus, the synchronous delivery mode was considered as one of this study’s assets. Future research on DRA telerehabilitation would benefit from further exploring synchronous models, whilst asynchronous models are subject to further investigation to enhance adherence and participation.

Overall, this study has significant clinical and research implications. It shows that therapeutic exercise provided via telerehabilitation holds significant clinical value for women with DRA, as it can help overcome time and mobility constraints. It offers a flexible, high-quality alternative when in-person participation is not feasible, while contributing to improvements not only in clinical parameters but also in psychological well-being. It also offers insights into different modes of telerehabilitation, highlighting that blended delivery methods might be both feasible and acceptable for these populations. However, synchronous methods may be more effective in promoting long-term adherence. In contrast, asynchronous telerehabilitation may be less effective when used alone, as integrating synchronous components appears essential for maintaining participant engagement.

However, while this study showed high satisfaction and preliminary clinical benefits, the cost-effectiveness of telerehabilitation for DRA remains underexplored. Telerehabilitation may reduce travel or childcare-related costs, but it also demands digital resources, and infrastructure. Future studies should consider evaluating cost-effectiveness to determine whether such interventions are economically sustainable.

This study also offers valuable insights into DRA rehabilitation, as it provides a comprehensive and progressive therapeutic exercise approach targeting all key muscles and focusing on functional outcomes. The 12-week program was designed in alignment with current guidelines for postpartum pelvic floor rehabilitation, DRA management, and general postpartum exercise, reflecting recent research recommendations that identify this duration as effective for achieving clinical changes [9,19,40,41,42]. The present study also offers insights into usability and patient satisfaction with this type of intervention, while using a validated questionnaire. It lays important groundwork for future high-quality RCTs focused on DRA telerehabilitation during the postpartum period. Moreover, it sets the stage for future research exploring the long-term effects of such interventions, their cost-effectiveness, and the potential technical enhancements that could improve the user experience.

### Limitations

Despite its important findings, this study was a small-sized pilot, which limits the generalizability of its findings. The absence of a control group limits the ability to draw definitive conclusions about the efficacy in impacting clinical outcomes. However, as this was an exploratory pilot assessing primarily the feasibility and acceptability of blended (synchronous and asynchronous) telerehabilitation for postpartum populations, future randomized controlled trials are required to confirm these preliminary findings. Synchronous sessions were rated highly in terms of satisfaction and usability, while adherence to asynchronous sessions was low. This suggests that synchronous supervision may be more suitable for postpartum women, while asynchronous components may need substantial adaptation to address their specific barriers. Future comparative or hybrid design trials are required to determine the optimal balance between synchronous and asynchronous delivery for this population. Furthermore, due to the single-arm pilot design, this study could not provide direct comparisons of telerehabilitation with in-person care. Thus, future randomized controlled trials are needed to determine the comparative effectiveness of telerehabilitation versus traditional rehabilitation.

Another limitation was recruitment methods (through posters and leaflets), which may have led to potential sampling bias due to a self-selected and perhaps more motivated participant group. However, this recruitment strategy was used because our university laboratory lacks a direct clinical referral pathway, and DRA cases are not frequently referred to physiotherapy in our region. The fact that one of the assessors was not blinded to the assessments of IRD and endurance tests could also potentially have introduced bias. However, due to the small pilot design and resource limitations, this approach was inevitable at the time. Furthermore, the exclusion of participants with a BMI over 30 kg/m^2^ might have limited the generalizability of the results; however, this selection was based on previously reported imaging difficulties associated with higher abdominal adiposity, which can affect measurement consistency [43,44,45].

Additionally, a substantial number of women (n = 25) who initially expressed interest did not proceed to baseline assessment, and five participants discontinued the intervention, mainly because of new pregnancies, and to a lesser extent due to health issues or competing family/work obligations. These factors highlight the challenges of recruiting and retaining postpartum women in rehabilitation trials and should be strongly considered in the design of future studies.

Another limitation of this study is that multiple secondary outcomes were tested without multiplicity correction, thus increasing the risk of Type I error. Therefore, the results for secondary outcomes should be considered exploratory and be interpreted with caution.

Adherence tracking in this study relied on self-reported data for the asynchronous (video-recorded) sessions, unlike other studies [12], where researchers monitored user engagement through platform analytics. In our case, such monitoring was not possible due to software limitations, which may have introduced recall and social desirability bias. However, asynchronous rehabilitation constituted only 1/3 of the telerehabilitation program, and synchronous session adherence was objectively monitored by the therapists.

Although overall satisfaction with the program was high, a lower score in the “system reliability” subcategory of the TUQ might indicate the need for further technical improvements, such as improved connection stability or more effective error messaging and troubleshooting.

## 5. Conclusions

The findings of this pilot study indicate that a 12-week exercise program delivered via telerehabilitation through a blended synchronous and asynchronous mode can be a satisfactory and potentially effective option for women with DRA postpartum. Participants reported high usability and satisfaction with the program, appreciating its ease of use and the quality of interaction with therapists. Adherence rates were notably high, particularly with the synchronous telerehabilitation mode, and significant improvements were observed in key clinical outcomes, such as IRD, trunk muscle endurance, and body image. Thus, telerehabilitation appears to be a feasible, acceptable, and effective solution that meets the needs of this specific population, reducing or eliminating common participation barriers, such as time constraints and travel difficulties. Asynchronous modes of telerehabilitation are not ideally recommended to be delivered on their own, without some form of synchronous methods to retain adherence.

## Figures and Tables

**Table 1:** Participant demographics.

**Category**	**Mean (SD)**
Age (years)	37.54 (5.5)
Height (cm)	165.31 (4.6)
Weight (kg)	63.54 (9.5)
BMI (kg/m^2^)	23.25 (3.4)
Time postpartum (months)	26.69 (21.1)
**Types and number of births**	**Percentage (Frequency)**
Primiparous (n = 5)	38.46%
-vaginal birth (n = 2)	40%
-cesarean (n = 3)	60%
Multiparous (n = 8)	61.54%
-vaginal birth (n = 5)	62.5%
-cesarean (n = 3)	37.5%

**Table 2:** Telehealth Usability Questionnaire (TUQ_Greek) sample scores.

TUQ_Greek Factors	Mean (SD)	Median	Min–Max	IQR *
Usefulness (Items 1–3)	6.10 (0.98)	6.33	12–21	1.83
Ease of Use and Learnability (Items 4–6)	6.77 (0.44)	7.00	17–21	0.33
Interface Quality (Items 7–10)	6.63 (0.57)	6.75	20–28	0.50
Interaction Quality (Items 11–14)	6.54 (0.59)	6.75	21–28	0.75
Reliability (Items 15–17)	5.00 (1.56)	5.33	5–21	2.34
Satisfaction and Future Use (Items 18–21)	6.65 (0.54)	7.00	21–28	0.62
Overall TUQ_Greek (total score)	6.28 (0.60)	6.26	104–147	0.87

* IQR = interquartile range.

**Table 3:** Measurements of inter-recti distance (IRD) and body image (BISS_Greek) scores.

Outcome	Pre-Intervention	Post-Intervention	Mean Difference (95% CI)	*p*-Value	Effect Sizes (Cohen’s d)	95% CI (Lower–Upper)
	Mean (SD)					
IRD—2 cm below umbilicus	2.60 (0.93)	2.39 (0.74)	0.21 (−0.24 to 0.66)	0.321	0.30	−0.29 to 0.87
IRD—2 cm above umbilicus	3.70 (0.76)	3.40 (0.81)	0.29 (0.12 to 0.47)	* 0.004	1.00	0.31 to 1.66
IRD—5 cm above umbilicus	3.31 (0.98)	2.97 (0.88)	0.33 (0.08 to 0.58)	* 0.013	0.81	0.16 to 1.42
BISS_Greek	5.69 (1.84)	6.64 (1.15)	−0.95 (−1.75 to −0.16)	* 0.023	−0.73	−1.33 to −0.10

* Statistically significant changes.

**Table 4:** Trunk endurance test scores.

Test	Baseline	Week 4	Week 8	Week 12	Significant Pairwise Comparisons (Bonferroni-Adjusted)/*p*-Value (Repeated Measures ANOVA)	Partial η^2^
	Mean (SD) in sec					
Curl-up	119.06 (84.5)	99.10 (57.4)	168.68 (97.5)	203.97 (114.4)	p = 0.134 (No significant pairwise comparisons)	0.488
Front Plank	46.57 (29.4)	60.99 (27.6)	72.60 (28.7)	80.41 (28.6)	p = 0.113 (No significant pairwise comparisons)	0.441
Right Side Plank	31.73 (17.3)	37.66 (12.5)	41.32 (8.3)	51.21 (17.4)	Baseline< Week 12 (*p* = 0.029 *)	0.483
Left Side Plank	28.26 (18.2)	35.36 (15.3)	42.16 (13.0)	49.45 (15.1)	Baseline < Week 12 (*p* = 0.028 *) Week 4 < Week 12 (*p* = 0.010 *) Week 8 < Week 12 (*p* = 0.028 *)	0.562
McGill’s Trunk Flexor Endurance	62.00 (28.0)	92.01 (28.8)	117.45 (60.9)	128.93 (61.2)	Baseline < Week 4 (*p* = 0.036 *) Baseline < Week 12 (*p* = 0.023 *)	0.477

* significant pairwise comparisons.

## Data Availability

The original contributions presented in this study are included in the article/Supplementary Material. Further inquiries can be directed to the corresponding author.

## Supplementary Materials

The following supporting information can be downloaded at: https://www.mdpi.com/article/10.3390/healthcare13172224/s1, Table S1. Examples of progressive stabilization exercises practised throughout the program. Table S2. Thematic categories and representative participant responses regarding the use of telerehabilitation (Zoom) compared with in-person sessions. Figure S1. Ultrasound image showing the inter-recti distance (IRD) measured in a postpartum woman during baseline evaluation. The image was acquired with the participant in a supine, relaxed position. The measurement point was located 2 cm above the upper border of the umbilicus. Caliper placement was performed manually, and the IRD (3.35 cm in this example) was calculated by the system. RA indicates the rectus abdominis muscle bellies.

## Abbreviations

The following abbreviations are used in this manuscript:

DRA	Diastasis recti abdominis
TUQ	Telehealth Usability Questionnaire
IRD	Inter-recti distance
BISS	Body Image States Scale
