# Supplementary material for: Feasibility and Preliminary Efficacy of a Telerehabilitation Intervention for Diastasis Recti Abdominis—A Pilot Study
Source: Healthcare (Basel). 2025 Sep 5;13(17):2224. doi: 10.3390/healthcare13172224 (PMC12427742; doi:10.3390/healthcare13172224)
Supplement: Supplementary file 1 [file healthcare-13-02224-s001.zip › healthcare-3784002-supplementary.pdf]

Supplementary Material

Table S1. Examples of progressive stabilization exercises practised throughout the program.

**Phase (Weeks)**	**Position**	**Focus**	**Example Exercise (repetitions)**	**Progression**	**Pelvic Floor Muscle Contractions**
Weeks 1–4	Supine	Core activation, pelvic control, abdominal exercises	Leg raises variations<br>Bridge variations<br>Curl-up variations<br><br>(4-8 repetitions progressively)	Week 2<br>Progress reps (from 4 to 6) **if no**:<br>- bulging<br>- Valsalva<br>- loss of pelvic floor muscle contraction<br>- loss of pelvic control<br>- subjective difficulty<br>-RPE >4<br><br>Week 4<br>Progress reps (from 6 to 8) **if no**:<br>- bulging<br>- Valsalva<br>- loss of pelvic floor muscle contraction<br>- rib flare<br>- trunk rotation<br>-pelvic tilt or drop<br>-RPE>5	Contraction hold:6 sec<br>"Slow" repetitions: 6<br>"Fast" repetitions: 6
	Side-lying	Core activation, Hip abductor exercises, trunk endurance	Leg raises variations<br>Side plank variations<br><br>(4-8 repetitions progressively)		
	Prone	Core Activation, Spinal extensor endurance<br>Thoracic and pelvic control	Thoracic extensions<br>Leg extensions<br>Opposite arm–leg lifts<br><br>(4-8 repetitions progressively)		
	Quadruped	Core activation/stability with limb movement	Static abdominal contractions<br>Bird-dog<br>Plank variations		

			(4-8 repetitions progressively)		
	Sitting	Core activation, abdominal exercises	Pelvic curl and twist variations<br><br>(4-8 repetitions progressively)		
Weeks 5–8	Supine	Core activation, Increased load & ROM	Double leg raises (small lever arms)<br>Single-leg bridges<br>Criss-cross abs<br><br>(6-10 repetitions progressively)	<u>Week 6</u><br>Progress reps from 8 to 10 **if no**:<br>- bulging<br>- Valsalva<br>- loss of pelvic floor muscle contraction<br>- rib flare<br>- external oblique substitution<br>- loss of pelvic and/or spine control<br>-RPE>5<br><br><u>Week 8</u><br>Progress exercise difficulty **if no**:<br>-loss of abdominal and pelvic floor control<br>-compensatory movements<br>- knee valgus/instability<br><br>If RPE ≤ 5 at 10 reps, increase load/lever arm, then reset to 6 reps. If RPE is 6 at 8 reps, stay at 8 until it drops.	Contraction hold:6 sec<br>“Slow” repetitions: 8-10<br>“Fast” repetitions: 8-10
	Side-lying	Core activation under increased load	Side plank on feet/+leg movement<br><br>(6-10 repetitions progressively)		
	Prone	Core Activation, Dynamic thoracic and pelvic control with limb extension	Opposite arm–leg lifts<br><br>(6-10 repetitions progressively)		
	Quadruped	Core activation, Static-dynamic transitions and increased load	Plank with leg lifts<br><br>(6-10 repetitions progressively)		

	Sitting/Standing	Core activation, Multi-planar control<br>Added resistance	Side bending/ trunk twists (+elastic bands) Curls and twists (+weights)<br>Squat progressions<br><br>(6-10 repetitions progressively)		
Weeks 9–12	Supine	Core activation Advanced control (increased lever arms)	Double leg raises (increased lever arms)<br><br>(8-10 repetitions)	Week 10<br>Maintain RPE ≤ 6.<br>If RPE ≤ 5 at 10 reps, progress exercise difficulty (resistance, longer lever arm), reset to 8 reps.	Contraction hold:8-10 sec<br>“Slow” repetitions: 10<br>“Fast” repetitions: 10
	Side-lying	Core activation Unstable & rotational loading	Side plank with trunk rotation<br><br>(8-10 repetitions)		
	Quadruped	Core activation Dynamic control	Push-up progressions<br><br>(8-10 repetitions)		
	Sitting/Standing	Core activation under high load and high impact exercises	Side bending/ trunk twists (+elastic bands/weight)<br>Squat progressions (+ weights) Squat jumps<br>Lunge variations<br>Kneeling twists with bands<br><br>(8-10 repetitions)		

Table S2. Thematic categories and representative participant responses regarding the use of telerehabilitation (Zoom) compared with in-person sessions.

Response Category	Representative Responses
**Zoom as an effective alternative when in-person sessions were not feasible**	• "Not as effective as in-person, but when in-person therapy was not possible, it served very satisfactorily."<br>• "Zoom was very helpful for adherence to exercises, because often some family issue would come up and I couldn't attend."<br>• "The Zoom program covered me to a large extent, because otherwise I would not have been able to attend therapy with three children."
**Comparable support and guidance between Zoom and in-person sessions**	• "It was almost at the same level. The only limitation was that the therapist could not demonstrate directly on your body; however, the clear instructions compensated for this."<br>• "I believe there was no communication problem compared to in-person. It was equivalent."<br>• "The level of support/guidance via Zoom was truly just as good and of high quality as in-person."
**Perceived advantages of in-person sessions**	• "In-person sessions are always more complete in terms of support and guidance."<br>• "In-person is more pleasant and interesting, whereas remote is more convenient for me."<br>• "In-person is definitely better, because you can get direct feedback from the clinician."
**Preference for a combined approach (blended telerehabilitation and in-person)**	• "Ultimately, telerehabilitation is helpful if no other solution exists or in combination with in-person sessions."<br>• "Both offered the same level of support. Remote covered one aspect and in-person another."

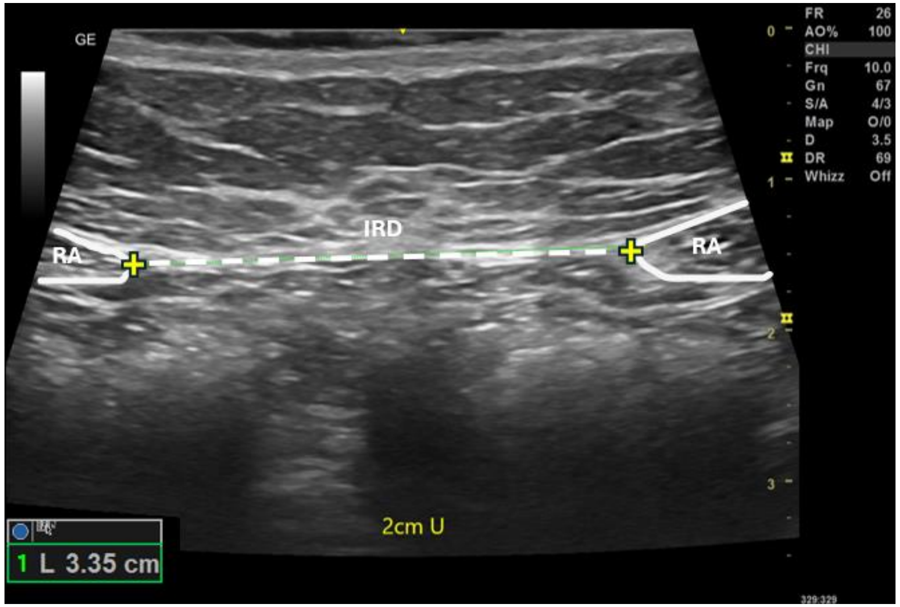

Figure S1. Ultrasound image showing the inter-recti distance (IRD) measured in a postpartum woman during baseline evaluation. The image was acquired with the participant in a supine, relaxed position. The measurement point was located 2 cm above the upper border of the umbilicus. Caliper placement was performed manually, and the IRD (3.35 cm in this example) was calculated by the system. RA indicates the rectus abdominis muscle bellies.
